# Protocol for constructing asymmetric triple-atoms supported on nitrogen-doped carbon nanotubes via atomic layer deposition

**DOI:** 10.1016/j.xpro.2025.104005

**Published:** 2025-07-31

**Authors:** Jingyan Zhang, Zhongxin Song, Xiaozhang Yao, Yi Guan, Ziwei Huo, Ning Chen, Lei Zhang, Xueliang Sun

**Affiliations:** 1 Department of Mechanical and Materials Engineering, University of Western Ontario, London, ON N6A 5B9, Canada; 2 Eastern Institute for Advanced Study, Eastern Institute of Technology, Ningbo, Zhejiang 3150200, P.R. China; 3 College of Chemistry and Environmental Engineering, Shenzhen University, Shenzhen 518060, P.R. China; 4 Science Division, Canadian Light Source Inc., 44 Innovation Boulevard, Saskatoon, SK S7N 2V3, Canada

**Keywords:** Energy, Chemistry, Material sciences

## Abstract

The precise construction of highly efficient triple-atom catalysts (TACs) remains a significant challenge. Here, we present a protocol for constructing asymmetric Pt-Ru-Co triple-atoms (TAs) supported on nitrogen-doped carbon nanotubes (NCNTs) via selective atomic layer deposition (ALD) technology. We describe steps for synthesizing the NCNT substrate, deposition of Pt-Ru-Co TAs, and characterizing the materials. We then detail procedures for preparing the electrolyte solution and working electrode followed by electrochemistry measurements for hydrogen evolution and hydrogen oxidative reactions with a typical three-electrode system.

For complete details on the use and execution of this protocol, please refer to Zhang et al.^1^

## Highlights

• Instructions for the synthesis of nitrogen-doped carbon nanotubes
• Atomic layer deposition procedures for Pt-Ru-Co triple-atoms
• Steps for structure characterization of triple-atom catalysts
• Guidance on electrocatalytic performance evaluation of catalysts

## Abstract (editor highlights)

Publisher’s note: Undertaking any experimental protocol requires adherence to local institutional guidelines for laboratory safety and ethics.

## Graphical abstract

**Figure:**
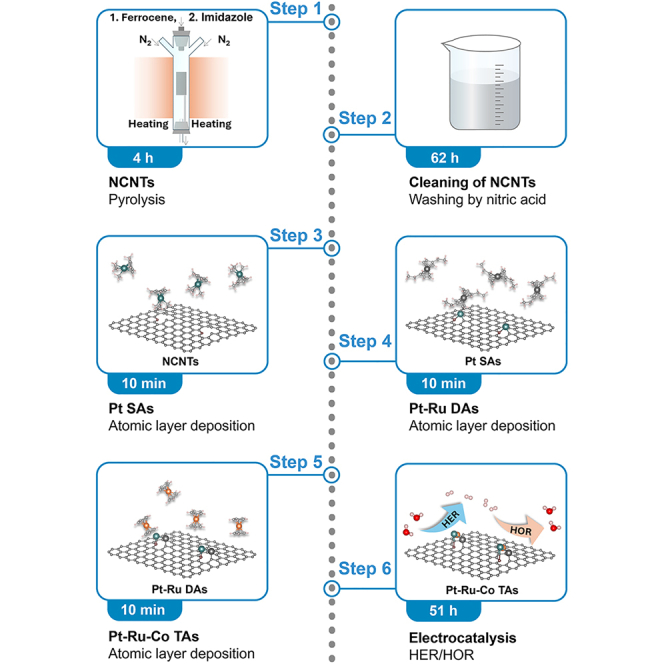

## Before you begin

Atomically dispersed catalysts (ADCs), including single-atom catalysts (SACs), dual-atom catalysts (DACs), and triple-atom catalysts (TACs), have garnered significant attention due to their unique electronic structures, high atomic utilization, and tunable catalytic properties. SACs, in particular, have demonstrated exceptional performance in various catalytic reactions.^2^^,^^3^^,^^4^ By anchoring isolated metal atoms onto suitable supports, SACs can achieve superior activity and selectivity. However, their practical applications are hindered by challenges such as low active site density, instability due to metal atom migration and aggregation, and strong dependence on the local coordination environment.^5^^,^^6^^,^^7^ Developing ADCs with an increased number of atomically dispersed metal sites and enhancing their catalytic activities are essential for their practical applications.

Inheriting the advantages of typical SACs, DACs and TACs have demonstrated outstanding performance in electrocatalytic applications due to synergistic interactions between adjacent metal atoms. Compared to DACs, TACs offer greater flexibility in electronic structure modulation and optimized adsorption of reaction intermediates, resulting in superior catalytic activity. For instance, PtFeCo/NC triple-atoms (TAs) exhibit more than five times the mass activity at 0.85 V compared to their binary counterparts in the OER, where Pt effectively modulates the adsorption strength of intermediates on both Fe–N_4_ and Co–N_4_ sites.^8^ Similarly, in atomically dispersed ZnCoFe TAs, Zn and Fe atoms regulate the electronic structure of Co active sites, optimizing the *d*-band center and weakening Co–O binding energy, thereby enhancing catalytic performance in oxygen-related reactions.^9^ Despite their promising potential, the precise synthesis of TACs remains a significant challenge. The traditional pyrolysis approach often leads to uncontrolled metal dispersion and aggregation, making it difficult to achieve precise atomic configurations. It remains challenging to accurately control and rationally design TACs.

Atomic layer deposition (ALD) has emerged as a powerful technique for the fabrication of ADCs. As a sequential self-limiting process, ALD prevents excessive adsorption of reaction precursors on the substrate surface, ensuring the controlled formation of atomically dispersed active sites. By optimizing ALD conditions, subsequent metal atoms can be selectively anchored around previously deposited atoms, enabling precise control over atomic configurations and metal coordination environments. In 2019, our group successfully synthesized bimetallic Pt-Ru dimer structures using ALD, demonstrating that Ru atoms could be precisely deposited onto Pt atoms to form well-defined dimers.^10^ Moreover, ALD offers additional advantages such as short processing time, low energy consumption, and excellent scalability, making it a highly promising and practical approach for the large-scale, precise fabrication of TACs.

This protocol presents detailed preparation for asymmetric Pt-Ru-Co triple-atoms (TAs) supported on NCNTs via selective ALD technology. By optimizing deposition conditions, stable and well-defined heterogeneous Pt-Ru-Co TAs configurations are achieved. The synthesis of NCNTs substrate is also described. The structure of TAs was identified by the aberration-corrected high-angle annular darkfield scanning transmission electron microscopy (HAADF-STEM). X-ray absorption spectroscopy (XAS) analysis reveals the electronic interactions between the heterogeneous atoms in Pt-Ru-Co TAs. Experimentally, the Pt-Ru-Co TAs show better catalytic performance than Pt single atoms (SAs) and Pt-Ru dual-atoms (DAs) catalysts in the electrocatalytic hydrogen evolution reaction (HER) and hydrogen oxidative reaction (HOR). This protocol offers a powerful strategy for fabricating advanced TACs.

## Key resources table

**Table:** 

REAGENT or RESOURCE	SOURCE	IDENTIFIER
**Chemicals, peptides, and recombinant proteins**		

Imidazole	Sigma-Aldrich	CAS No. 288-32-4
Ferrocene	Sigma-Aldrich	CAS No. 102-54-5
Sulfuric acid (ACS reagent, 95.0%–98.0%)	Sigma-Aldrich	CAS No. 7664-93-9
Nitric acid (ACS reagent, 70%)	Sigma-Aldrich	CAS No. 7697-37-2
Trimethyl(methylcyclopentadienyl)-platinum (IV) (MeCpPtMe_3_)	Strem Catalog	SKU No. 94442-22-5
Bis(ethylcyclopentadienyl)ruthenium(II)	Strem Catalog	SKU No. 44-0040
Cobaltocene, 98%	Strem Catalog	CAS No. 1277-43-6
Nafion (Nafion 117 containing solution) 5% in a mixture of lower aliphatic alcohols and water	Sigma-Aldrich	CAS No. 31175-20-9
Isopropyl alcohol (HPLC Grade, purity ≥99.7%)	Sigma-Aldrich	CAS no. 67-63-0
Deionized water (18.25 MΩ cm)	Made by the pure water machine	N/A
Perchloric acid (ACS reagent, 60%)	Sigma-Aldrich	CAS no. 7601-90-3
N_2_ cylinder (5.0 purity)	Linda	CAS no. 7727-37-9
H_2_ cylinder (5.0 purity)	Linda	CAS no. 333-74-0
O_2_ cylinder (5.0 purity)	Linda	CAS no. 7782-44-7

**Software and algorithms**		

Origin	OriginLab	https://www.originlab.com/
Savannah (ALD deposition software)	Veeco	N/A
GPES manager (Autolab software)	N/A	N/A
Athena	Demeter	Version 0.9.26
XPSPEAK	Wise Solutions	Version 4.1

**Other**		

Ultrasonic atomizer	Sonics & Materials Inc., CT, USA	VCX-134ATDP 40kHz
Syringe pump	Sigma-Aldrich	KDS Legato™ 185 Syringe Pump
Ceramic boat, 95 mm	Alibaba	https://www.alibaba.com/
High-speed centrifuge	Hettich	Universal 320
Pure water machine	ULUPURE	UPH-II-10T
Atomic layer deposition	Ultratech	N/A
Scanning electron microscope	Hitachi	S-4800 field-emission
Conductive double sided carbon tape, 8 mm (conductive resistivity: 50 ohm/sq. inch, thickness: 0.16 mm, pad: 0.07 mm)	Fisher scientific	Catalog No.50-285-81
Transmission electron microscopy	Thermo Fisher Scientific	Talos 200X
Copper grid (lacey formvar/carbon, 200 mesh, copper)	Ted Pella, Inc.	Prod. # 01824
Atomic-resolution high-angle annular dark-field scanning transmission electron microscopy	Thermo Fisher Scientific	FEI Themis, 300 kV
X-ray absorption spectroscopy	Canadian Light Source	061D Superconducting Wiggler, HXMA
X-ray photoelectron spectroscopy	Kratos Analytical	Axis Ultra
Inductively coupled plasma optical emission spectrometer	Agilent Technologies	5110 ICP-OES
Analytical balance	OHAUS	PX124ZH
Ultrasonic cleaning machine	Prima	PM2-600TD
Blast drying oven	INTSUPERMAI	N/A
Autolab potentiostat/galvanostat	Metrohm	PGSTAT302
Modulated speed rotator	Pine Instrument Company	MSRX Speed Control

## Materials and equipment

• The ultrasonic atomizer and syringe pump (Sigma-Aldrich) were applied for the synthesis of NCNTs. A ceramic boat was used as the substrate for the growth of NCNTs.
• High-speed centrifuge (Hettich) was used for the cleaning and separation of NCNTs.
• The pure water machine (ULUPURE) was used to produce deionized water.
• Atomic layer deposition (Ultratech, simple ALD) equipment was used for preparation of triple-atoms catalysts.
• Scanning electron microscope (SEM) images were collected on Hitachi S-4800 field-emission SEM equipment at an acceleration voltage of 5 kV.
• Conductive double sided carbon tape was used to adhere catalysts onto the sample stage for SEM test.
• Transmission electron microscopy (TEM, Thermo Scientific Talos 200X) equipped with four in-column SDD Super-X detectors for energy dispersive X-ray spectroscopy (EDS) was used to collect the elements mapping.
• Copper grids were used to support the catalyst for TEM test.
• Atomic-resolution high-angle annular dark-field scanning transmission electron microscopy (HAADF-STEM) images of atomic structure were carried out on a double spherical aberration-corrected FEI Themis microscope operated at 300 kV.
• X-ray absorption spectroscopy (XAS) measurements were carried out on the 061D superconducting wiggler at the hard X-ray microanalysis (HXMA) beamline of Canadian Light Source (CLS). A Si (111) double-crystal monochromator was used to filter the X-ray beam.
• X-ray photoelectron spectroscopy (XPS, Kratos Axis Ultra) was applied to explore the surface oxidation state and electronic structure of different elements in catalysts. The binding energy of C 1s used for calibration is 284.6 eV.
• Inductively coupled plasma optical emission spectrometer (ICP-OES) was used to analyze the metal loadings.
• The analytical balance (OHAUS) was used to measure the mass of the catalyst.
• An ultrasonic cleaning machine was used to disperse the catalysts in deionized water to form homogeneous ink.
• The blast drying oven was applied to dry NCNTs after cleaning and electrodes after loading catalysts.
• The autolab potentiostat/galvanostat (Metrohm) was utilized for all the electrochemical experiments.
• A modulated speed rotator (MSR, Pine) was applied to control the rotation rate of electrode during hydrogen oxidation reaction.

## Step-by-step method details

### Synthesis of NCNT substrate

**Timing: 66.5 h**

**Figure 1:**
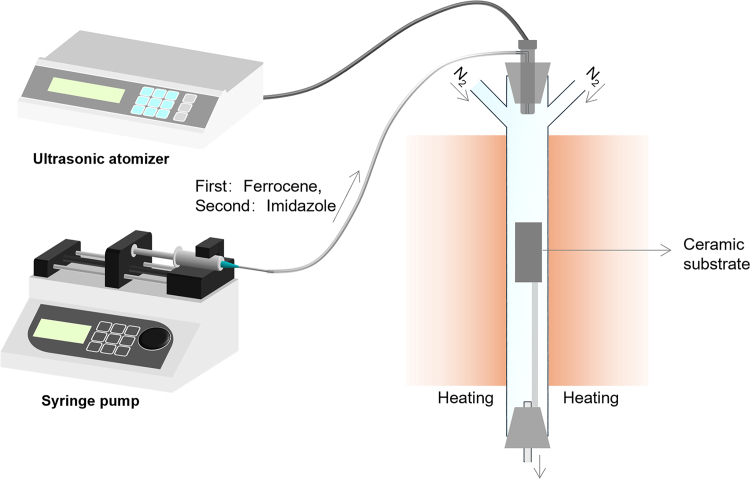
Schematic diagram of NCNTs synthesis

NCNTs were fabricated by ultrasonic spray pyrolysis approach, which were used as support material for ALD experiments. The detailed process is below and illustrated in Figure 1.

1. Preparation of NCNTs (4 h).
  ***Note:*** Ferrocene solution and imidazole solution can be separately kept in volumetric flask.
  a. Add 40.0 mL of acetonitrile and 0.8 g of ferrocene to a beaker. Stir it with a magnetic stirrer until it dissolves.
  b. Then, add 40.0 mL of acetonitrile and 8.0 g of imidazole to a beaker. Stir it with a magnetic stirrer until it dissolves.
  c. Use clean copper wire to fix a 95 mm rough ceramic boat (with a hole at the bottom) at the center of a custom-made quartz tube, serving as the growth substrate.
  d. Carefully place the custom-made quartz tube into the vertical tube furnace.
  e. Install an atomizer to the upper end of the quartz tube.
  f. Introduce high-purity nitrogen into both sides of tube with a nitrogen flow rate of 70–80 sccm for each side.
  g. Turn on the tube furnace and heat it to 850°C under the control of the temperature program
  h. Inject 2 mL ferrocene solution into the injector (Injector A) and connect the injector with the plastic tube of the atomizer, through which the solution will flow in.
  i. Install the injector on a syringe pump.
  j. Set the infuse rate as 0.33 mL/min.
  k. Turn on the atomizer and start syringe pump.
  l. Turn off the syringe pump once all the solution in Injector A has flowed out.
  m. Inject 9 mL of imidazole solution into another injector (Injector B), Connect the plastic tube of the atomizer to injector B, and then install injector B onto the syringe pump.
  n. Turn on the syringe pump.
  o. Turn off the syringe pump and atomizer once all the solution in Injector B has flown out.
  p. Then, turn off the furnace.
  q. After the temperature of the furnace cools down to room temperature, turn off the nitrogen gas and take the samples out.
  r. Finally, collect black NCNTs from ceramic substrate.
    **CRITICAL:** The synthesis experiment should be performed in a fume hood because some organic byproducts are generated in the process.
    ***Note:*** Make the nitrogen flow rate of both sides same to maintain a stable and uniform gas flow. Do not decrease the flow rate of nitrogen, as the high gas flow rate ensures efficient transport of the catalyst ferrocene and reactant imidazole into the reaction zone. The infuse rate of ferrocene and imidazole should be kept at 0.33 mL/min. An excessively high flow rate can lead to non-uniform growth size of carbon nanotubes.
2. Preparation of 2.0 mol/L nitric acid solution (30 min).
  a. Measure 31.5 mL of concentrated nitric acid (HNO_3_) using a graduated cylinder.
  b. Add about 100 mL of deionized water into a 250 mL volumetric flask.
  c. Slowly add the measured HNO_3_ into the flask containing water.
  d. Swirl the flask gently to mix the solution.
  e. After cooling, fill up the flask to the 250 mL mark with deionized water.
  f. Cap and mix well by inverting the flask several times.

**CRITICAL:** Wear appropriate personal protective equipment (PPE), including gloves, lab coat, and safety goggles. Work under a fume hood.

3. Cleaning of NCNTs (62 h).
  a. Add the carbon nanotubes into the glass beaker.
  b. Take 100 mL of 2 M nitric acid to a glass beaker.
  c. Cover the beaker with an acid-resistant lid.
  d. Place the beaker in a fume hood for 48 h.
  e. Transfer the mixture to an acid-resistant 50 mL centrifuge tube to centrifuge it at 7155 g (8000 rpm).
  f. Wash and centrifuge NCNTs eight times using deionized water until the supernatant is neutral.
  g. Dry the NCNTs in a vacuum oven for 12 h at temperature of 60 °C. The final yield of NCNTs is approximately 50 mg.

**CRITICAL:** Wear appropriate PPE. Work under a fume hood. Always balance the centrifuge before operation. Ensure that tubes placed opposite each other have the same type, volume, and total mass (including the tube and contents).

### Deposition of Pt-Ru-Co TAs

**Timing: 2.5 h**

**Figure 2:**
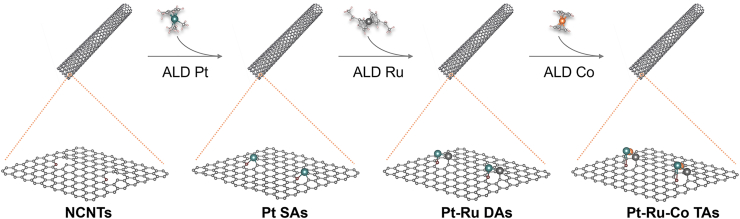
The digital photos of ALD equipment and the control software for ALD

Pt-Ru-Co TAs were prepared by ALD technology. The detailed process is described below and the ALD equipment and corresponding control software for ALD process are shown in Figure 2.

4. Preparation of substrate for ALD (30 min).
  a. Weigh 30 mg of NCNTs and add 10 mL of anhydrous ethanol.
  b. Ultrasonicate the mixture at room temperature for 10 min.
  c. Evenly drop the mixture onto an aluminum foil with the area of 15×15 cm^2^.
  d. Dry NCNTs on aluminum foil for 10 min at 60°C.

***Note:*** Evenly drop-cast the NCNTs dispersion onto aluminum foil, ensuring the layer is not too thick. During drying, avoid high temperatures to prevent the NCNTs from detaching from the foil.

5. Preparation of the precursor cylinders (30 min).
  a. Place the precursor bottles, chemical reagents, and adjustable wrench into the glovebox.
  b. Purge and refill the glovebox with high-purity N_2_ three times to ensure an oxygen- and moisture-free environment.
  c. Use the wrench to carefully open the precursor bottles and position them upright on a stable support rack.
  d. Weigh out 200 mg of solid precursor (or 1 mL if in liquid form).
  e. Gently transfer the measured precursor into the corresponding precursor bottle.
  f. Tightly close the bottle cap using the wrench and make sure the valve on the bottle is securely closed.
  g. Close the caps of all chemical reagent containers.
  h. Remove the precursor bottles and tools from the glovebox.
  i. Repeat vacuum-purge cycles three times to restore the inert glovebox environment.
  j. Install the precursor bottles onto the ALD instrument and keep them ready for deposition.

***Note:*** Seal the precursors very well and store them in a cold storage compartment of refrigerator to maintain a low temperature after using them. Always keep the precursor bottle valves closed when not in operation.

6. Preparation of Pt SAs (10 min).
  a. Turn on the ALD instrument, vacuum system and open the valve of carrier gas N_2_.
  b. Heat and keep the deposition chamber to 250°C. Heat and keep the manifold and exhaust line at 110°C.
  c. Heat and keep the precursor trimethyl(methylcyclopentadienyl)-platinum (IV) (MeCpPtMe_3_) to 65°C.
  d. Vent the chamber, place the aluminum foil loaded with NCNTs in the deposition chamber, and pump the chamber.
  e. Set up the deposition recipe for Pt SAs (Shown in Table 1).
  f. Open the valve of Pt precursor.
  g. Then, start the recipe.
  h. Close the valve of precursor.
  i. Vent the chamber and retrieve the catalyst loaded on aluminum foil after completion. Collect these black materials and Pt SAs are obtained.

**CRITICAL:** Avoid the leaking and inhaling of precursors during ALD process. Always wear heat-insulation gloves when opening the reactor chamber. Check and ensure that a stable precursor vapor stream is established before starting the deposition process. Additionally, check the integrity and airtightness of all gas lines and the ALD chamber to avoid leaks during operation.

***Note:*** The deposition temperature should not be too high or too low, otherwise it will affect the metal loading and the dispersion of deposited atoms. 250°C is an empirically suitable deposition temperature for Pt SAs. The temperature of the manifold and exhaust line should be kept higher than that of the precursor to prevent condensation of the precursor. Deposition recipe can be saved and loaded next time when needs to use it.

7. Preparation of Pt-Ru DAs (10 min).
  a. Turn on the ALD instrument, vacuum system and open the valve of carrier gas N_2_.
  b. Heat and keep the deposition chamber to 270 °C. Heat and keep the manifold and exhaust line at 150°C.
  c. Heat and keep the bis(ethylcyclopentadienyl)ruthenium(II) precursor to 110°C.
  d. Vent the chamber, place the aluminum foil loaded with Pt SAs supported by NCNTs in the deposition chamber, and then pump the chamber.
  e. Set up the deposition procedure for Ru (Shown in Table 1).
  f. Open the valve of the Ru precursor.
  g. Start the recipe of Ru.
  h. Close the valve of precursor.
  i. Vent the chamber and retrieve the catalyst loaded on aluminum foil after completion. Collect these black materials and Pt-Ru DAs are obtained.

***Note:*** If the deposition of Ru is immediately after Pt deposition, the step “a” can be skipped, the Ru precursor can be pre-heated before starting the deposition to save some time and don’t forget to increase the temperature of chamber and manifold.

**Figure 3:**
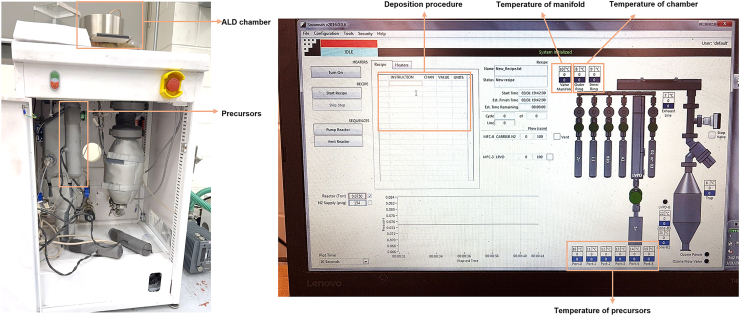
Schematic illustration of the synthesis process of Pt-Ru-Co TAs via the ALD technique Light gray: C atom, brown: N atom, gray blue: Pt atom, dark gray: Ru atom, orange: Co atom.

8. Preparation of Pt-Ru-Co TAs (10 min).
  a. Turn on the ALD instrument, vacuum system and open the valve of carrier gas N_2_.
  b. Heat and keep the deposition chamber to 250°C. Heat and keep the reactor inlets and manifold at 110°C.
  c. Heat and keep the cobaltocene (Co(Cp)_2_) precursor to 90°C.
  d. Vent the chamber, place the aluminum foil loaded with Pt-Ru DAs supported by NCNTs in the deposition chamber, and then pump the chamber.
  e. Set up the deposition recipe for Co (Shown in Table 1).
  f. Open the valve of Co precursor.
  g. Start the recipe for Co.
  h. Close the valve of precursor.
  i. Vent the chamber and retrieve the catalyst loaded on aluminum foil after completion. Collect these black materials and Pt-Ru-Co TAs are obtained. The final obtained Pt-Ru-Co TAs catalysts is about 20 mg. Schematic illustration of the synthesis process of Pt-Ru-Co TAs is shown in Figure 3.

**CRITICAL:** The deposition sequence of Pt-Ru-Co TAs must be Pt>Ru>Co, as only this sequence can form the desired the triple-atom structure.

***Note:*** If the deposition of Co is immediately after Ru deposition, the step “a” can be skipped, the Co precursor can be pre-heated before starting the deposition to save some time and don’t forget to decrease the temperature of chamber and manifold.

9. Preparation of Ru-Pt-Co (30 min).
  a. Turn on the ALD instrument, vacuum system, and open the valve of carrier gas N_2_.
  b. Heat and keep the deposition chamber at 270°C. Heat and keep the manifold and exhaust line at 150°C.
  c. Heat the precursors trimethyl(methylcyclopentadienyl)-platinum (IV) (MeCpPtMe_3_), bis(ethylcyclopentadienyl)ruthenium(II) and cobaltocene (Co(Cp)_2_) precursor to 90°C, 110°C and 65°C, respectively.
  d. Vent the chamber, place the aluminum foil loaded with NCNTs in the deposition chamber, and then pump the chamber.
  e. Open the valves of precursors.
  f. Set up and start the deposition recipe of Ru (Shown in Table 1).
  g. Set the temperature of the chamber to 250°C, the reactor inlets and manifold to 110°C.
  h. Wait for 10 min to allow the chamber and manifold to reach and stabilize at their respective target temperatures.
  i. Set up and start the deposition recipe of Pt and Co (Shown in Table 1).
  j. Close all the valves of precursors.
  k. Vent the chamber and retrieve the catalyst loaded on aluminum foil after completion. Collect these black materials and Ru-Pt-Co are obtained.

***Note:*** Since the optimal deposition temperatures for Pt and Co are relatively low, the chamber should be cooled down and stabilized at 250°C before initiating the deposition recipes for Pt and Co. The deposition recipe should be strictly designed according to the target deposition sequence.

10. Preparation of Co-Pt-Ru (30 min).
  a. Turn on the ALD instrument, vacuum system, and open the valve of carrier gas N2.
  b. Heat and keep the deposition chamber at 250°C. Heat and keep the reactor inlets and manifold at 110°C.
  c. Heat the precursors trimethyl(methylcyclopentadienyl)-platinum (IV) (MeCpPtMe3), bis(ethylcyclopentadienyl)ruthenium(II) and cobaltocene (Co(Cp)2) precursor to 90°C, 110°C and 65°C, respectively.
  d. Vent the chamber, place the aluminum foil loaded with NCNTs in the deposition chamber, and then pump the chamber.
  e. Open the valves of precursors.
  f. Set up the deposition recipe of Co and Pt (Shown in Table 1).
  g. Start the deposition recipe.
  h. Heat and keep the deposition chamber to 270°C. Heat and keep the manifold and exhaust line at 150°C.
  i. Set up and start the deposition recipe of Ru (Shown in Table 1).
  j. Close all the valves of precursors.
  k. Vent the chamber and retrieve the catalyst loaded on aluminum foil after completion. Collect these black materials and Co-Pt-Ru are obtained.

***Note:*** To ensure that Co is deposited before Pt, the sequence was arranged with Co preceding Pt in the deposition recipe. Since the optimal deposition temperature for Ru is higher, the chamber needs to be preheated to 270°C before introducing the Ru precursor.

**Table 1:** ALD recipes for Pt, Ru, and Co

Deposition	Instruction	Channel	Value (s)
Pt	Wait		20
	Pulse	1	10
	Wait		20
	Pulse	6	10
	Wait		60
Ru	Wait		20
	Pulse	2	10
	Wait		20
	Pulse	6	10
	Wait		60
Co	Wait		20
	Pulse	3	10
	Wait		20
	Pulse	6	10
	Wait		60

Here, channels 1, 2, and 3 are assigned to the Pt, Ru, and Co precursors, respectively, while channel 6 is used for O_2_ delivery.

### Material characterization

**Timing: 12 h**

**Figure 4:**
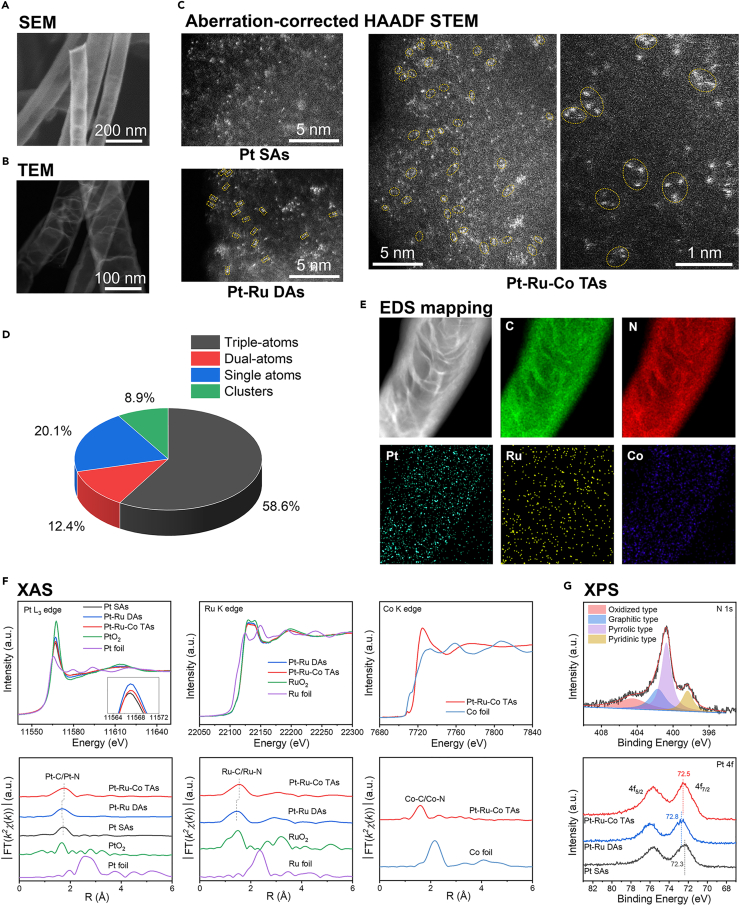
Material characterization (A) SEM image of synthesized NCNTs. (B) TEM image of synthesized NCNTs. (C) Aberration-corrected HADFF-TEM images of prepared Pt SAs, Pt-Ru DAs and Pt-Ru-Co TAs. The dual-atom structures in Pt-Ru DAs were marked by yellow dotted rectangles. The triple-atom structures in Pt-Ru-Co TAs were marked by yellow dotted circles. (D) Distribution pie chart of the ratio of triple-atoms, dual-atoms, single atoms and clusters in the as-prepared Pt-Ru-Co TAs. (E) EDS elemental mapping of Pt-Ru-Co TAs supported by NCNTs (C, N, Pt, Ru and Co). (F) XAS results of Pt SAs, Pt-Ru DAs, Pt-Ru-Co TAs and reference materials. (G) High-resolution N 1s spectrum of NCNTs and Pt 4f spectra of Pt SAs, Pt-Ru DAs, Pt-Ru-Co TAs and Co 2p spectrum of Pt-Ru-Co TAs. Figure reprinted with permission from Zhang et al.^1^

The metal loadings were analyzed by an inductively coupled plasma optical emission spectrometer (ICP-OES). The morphology and size of NCNTs was characterized by scanning electron microscope (SEM). The elements mapping was collected by using an analytical transmission electron microscopy (TEM) equipped with four in-column SDD Super-X detectors for energy dispersive X-ray spectroscopy (EDS). Atomic-resolution high-angle annular dark-field scanning transmission electron microscopy (HAADF-STEM) images were collected to observe the atomic structure of catalysts. X-ray photoelectron spectroscopy (XPS) was applied to explore the surface oxidation state and electronic structure of different elements in catalysts. These characterization results are shown in Figure 4.

11. The detailed preparation process of solutions for ICP test is described below. The results are shown in Table 2.
  a. Accurately weigh 5 mg catalyst containing single atoms supported on NCNTs into a clean 100 mL round-bottom flask.
  b. Add 18 mL concentrated hydrochloric acid (HCl) and 6 mL nitric acid (HNO_3_) to the flask containing the sample.
  c. Place the flask into a water bath equipped with a reflux condenser.
  d. Heat the water bath to 60°C and maintain reflux conditions for 10 h to ensure complete digestion of the metal species.
  e. Slowly add 76 mL of deionized water to the flask after the solution to cool to room temperature.
  f. Stir the solution gently with a magnetic stirrer for 20 min.
  g. Then, centrifuge it at 7155 g (8000 rpm) for 10 min and separate the supernatant. Take 5 mL of the supernatant and filter it using a 0.22 μm syringe filter to remove any residual carbon particles.
  h. Dilute the obtained solution with deionized water to a final volume of 10 mL and keep the final solution into acid-cleaned ICP sample vials.
  i. Label appropriately with sample ID, dilution factor, and digestion date. Store at room temperature until analysis. The ICP results are shown in Table 2.

**CRITICAL:** Wear appropriate PPE. This experiment should be performed under a fume hood. During water bath heating, water should be added to the bath at regular intervals to prevent dry heating and potential equipment damage.

***Note:*** To ensure complete dissolution of all metal single atoms in aqua regia, the water bath heating time should not be less than 10 h.

12. For SEM and XPS sample preparation, the catalysts need to be adhered onto the conductive double sided carbon tape.

***Note:*** For SEM analysis, the sample should be applied as a thin and uniform layer to ensure optimal imaging quality. For XPS measurements, a slightly thicker coating is acceptable.

13. Prepare the sample for EDS and HAADF-STEM test according to the following procedures.
  a. Disperse 1 mg catalysts powder in 30 mL ethanol aqueous solution and sonicate for 30 min to obtain a uniform suspension.
  b. Drop the suspension onto the copper grid, dry at 50°C, and examine the sample by TEM.

***Note:*** The solution can be dropped on copper mesh using a pipette. Apply one drop at a time and allow it to dry before adding the next. Typically, 1–2 drops in total are sufficient on a lacey copper grid, which allows for the observation of sample edges or suspended regions at high resolution, with reduced background interference.

**Table 2:** The weight contents of Pt, Ru, and Co elements in catalysts via ICP analysis

Catalysts	Pt (wt. %)	Ru (wt. %)	Co (wt. %)	Ratio of single atoms in catalysts
Pt SAs	1.02	–	–	–
Pt-Ru DAs	1.02	0.48	–	Pt : Ru=1 : 0.91
Pt-Ru-Co TAs	1.02	0.48	0.26	Pt : Ru : Co=1 : 0.91 : 0.84

### Electrochemical measurements

**Timing: 51 h**

**Figure 5:**
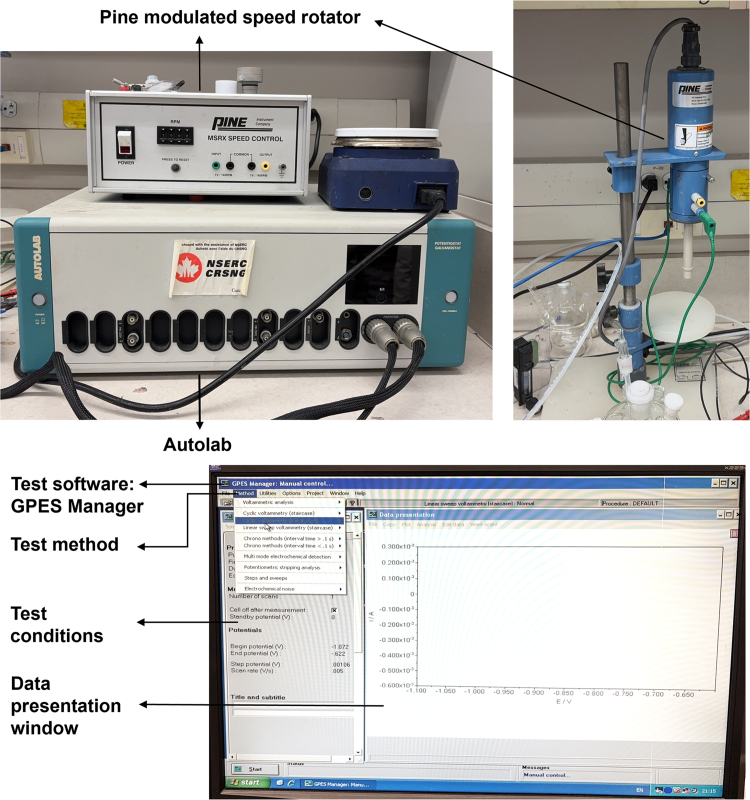
The digital photos of Autolab, speed rotator, and GPES software for electrochemical measurements of catalysts

**Figure 6:**
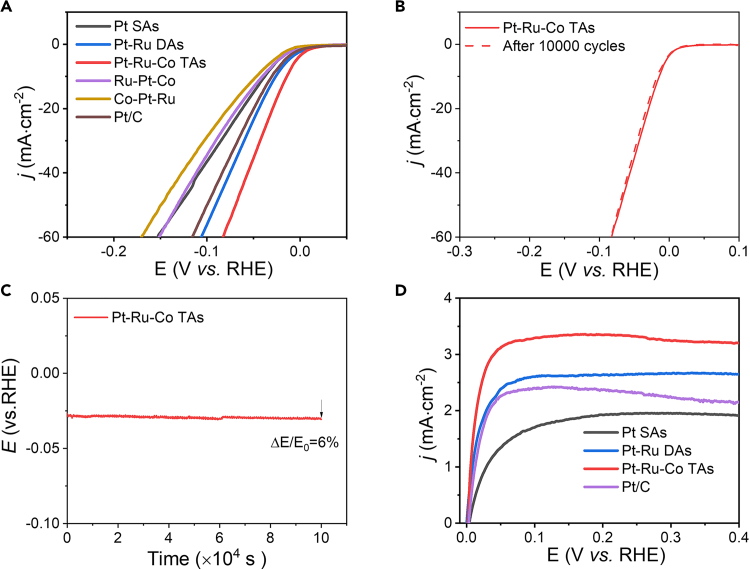
Electrochemical measurements (A) Polarization curves of catalysts for HER in 0.5 M H_2_SO_4_ solution. (B) Polarization curves of Pt-Ru-Co TAs before and after 10000 cyclic voltammetry tests. (C) Durability measurement of the Pt-Ru-Co TAs for HER. (D) Polarization curves of catalysts for HOR in 0.1 M HClO_4_ solution. Figure reprinted with permission from Zhang et al.^1^

In this step the catalyst ink was prepared and loaded onto a glassy carbon rotating-disk electrode (RDE, 5 mm in diameter), which is later applied as the working electrode. Besides, electrocatalytic HER and HOR performance were evaluated by a typical three-electrode system. The detailed process is below, the instruments used are shown in Figure 5 and the results are shown in Figure 6.

14. Preparation of the electrolyte solution (1 h).
  a. Preparation of 0.5 mol/L sulfuric acid solution.
    i. Add about 100 mL of deionized water into a 250 mL volumetric flask.
    ii. Measure 6.9 mL of concentrated sulfuric acid (H_2_SO_4_) using a graduated cylinder.
    iii. Slowly add the measured H_2_SO_4_ into the flask containing water.
    iv. Swirl the flask gently to mix the solution.
    v. After cooling, fill up the flask to the 250 mL mark with deionized water.
    vi. Cap and mix well by inverting the flask several times.
      **CRITICAL:** Wear appropriate PPE, including gloves, lab coat, and safety goggles. Work under a fume hood. The acid should be added gradually to the water to ensure safety and control of the reaction.
  b. Preparation of 0.2 mol/L perchloric acid solution.
    i. Add about 100 mL of deionized water into a 250 mL volumetric flask.
    ii. Measure 8.4 mL of concentrated perchloric acid (HClO_4_) using a graduated cylinder.
    iii. Slowly add the measured HClO_4_ into the flask containing water.
    iv. Swirl the flask gently to mix the solution.
    v. After cooling, fill up the flask to the 250 mL mark with deionized water.
    vi. Cap and mix well by inverting the flask several times.
      **CRITICAL:** Wear appropriate PPE, including gloves, lab coat, and safety goggles. Work under a fume hood. The acid should be added gradually to the water to ensure safety and control of the reaction.
15. Preparing the working electrode (1 h).
  a. Prepare the mixture solvent by blending 990.0 μL isopropanol, 990.0 μL of deionized water and 20.0 μL of 5% Nafion^T^ solution.
  b. Add 2.0 mg as-prepared catalysts into the mixture solvent.
  c. Sonicate the ink for 30 min at room temperature.
  d. Drop a total of 24, 15, 12, and 20 μL of catalyst ink for Pt SAs, Pt-Ru DAs, Pt-Ru-Co TAs, and Pt/C (40% Pt), respectively, onto separate electrodes with a diameter of 5 mm.
  e. Dry these electrodes at a temperature of 60°C.

***Note:*** Here, the overall metal loadings of the Pt SAs, Pt-Ru DAs, and Pt-Ru-Co TAs catalysts differ significantly. Therefore, the volume of catalyst ink deposited onto the glassy carbon electrode was adjusted accordingly to ensure comparable metal loading across different catalysts during electrochemical testing.

16. Electrochemistry measurements with a typical three-electrode system (49 h).
  a. Electrochemistry measurements for HER.
    i. Add 50.0 mL of 0.5 M H_2_SO_4_ into a 100.0 mL of the three-neck round bottom flask.
    ii. Insert the working electrode into the solution.
    iii. Insert the home-made reversible hydrogen electrode as a reference electrode and place the commercial Pt wire into the electrolyte as a counter electrode.
    iv. Purge the solution with argon for 30.0 min.
    v. Connect the electrodes with appropriate connectors of Autolab potentiostat. Use GPES manager software for data collection while using Autolab potentiostat.
    vi. Record polarization curve thorough the linear sweep Voltammetry (LSV) method.
    vii. Perform the cyclic voltammetry test for 10000 cycles. The voltage range is from −0.15 V to +0.4 V. The scan rate is 100 mV·s^−1^. The voltages are referenced to reversible hydrogen electrode (vs. RHE).
    viii. Record polarization curve again through the linear sweep Voltammetry (LSV) method after cyclic voltammetry (CV) test.
    ix. Change to another work electrode loaded catalysts.
    x. Perform the chronoamperometry test for 100000 s at the current of 10 mA·cm^−2^.
      ***Note:*** The reversible hydrogen electrode needs to be freshly prepared when the hydrogen bubble disappears. The preparation method involves connecting both the reference and counter electrodes to a platinum wire, with the reversible hydrogen electrode serving as the working electrode. A hydrogen evolution reaction is then carried out until a large hydrogen bubble forms inside the reversible hydrogen electrode.
  b. Electrochemistry measurements for HOR.
    i. Add 50.0 mL of 0.1 M HClO_4_ into a 100.0 mL of the three-neck round bottom flask
    ii. Insert the working electrode into the solution.
    iii. Insert the Ag/AgCl reference electrode and place the commercial Pt wire into the electrolyte as a counter electrode.
    iv. Purge the solution with hydrogen for 30.0 min and keep the hydrogen gas flow at 10 sccm.
    v. Set the rotation rate of round-disk electrode as 1600 rpm. Connect the electrodes with appropriate connectors of Autolab and record the polarization curves by LSV procedure. A scan rate of 5 mV·s^−1^.
      ***Note:*** Ensure continuous hydrogen flow throughout the test to maintain a hydrogen-saturated solution.
  c. Calculation of mass activities of catalysts.
    i. Calculate the catalysts loading (m_**1**_**,** mg) on the working electrode based on the volume of catalyst ink dropped.
      $$m_{1}=\frac{\text{Volume}\;\text{of}\;\text{dropped}\;\text{ink}\;\left(\mu L\right)}{2000\;\mu L\;\left(\text{the}\;\text{total}\;\text{volume}\;\text{of}\;\text{ink}\right)}\times 2\;mg$$
    ii. Calculate the metal loading (m_**2**_**,** mg) on the working electrode based on the metal content in each catalyst as determined by ICP analysis, which shows in Table 2. The metal content for commercial Pt/C is 40%.
      $$m_{2}=m_{1}\times \text{Metal}\;\text{wt}\%\;\left(\text{from}\;\text{ICP}\right)$$
    iii. Obtain the measured current (I, A) from the linear sweep voltammetry (LSV) test for the hydrogen evolution reaction (HER) or HER at a selected overpotential of 50 mV.
    iv. Calculate the mass activity (MA, A mg^−1^) at the overpotential of 50 mV by dividing the measured current by the corresponding metal loading:
      $$\text{MA}=m_{2}/I$$
      ***Note:*** For better accuracy, repeat the linear sweep voltammetry (LSV) test at least three times and calculate the average value. The mass activities results are shown in Table 3.

**Table 3:** The mass activities of Pt SAs, Pt-Ru DAs, Pt-Ru-Co TAs, and commercial Pt/C for HER and HOR

Catalysts	MA for HER at η=50 mV (A mg^−1^)	MA for HOR at η=50 mV (A mg^−1^)
Pt SAs	9.8	1.07
Pt-Ru DAs	20.5	2.08
Pt-Ru-Co TAs	32.9	2.89
Pt/C	0.5	0.05

## Expected outcomes

This protocol enables the construction of asymmetric heterogeneous Pt-Ru-Co TAs via a precisely controlled ALD technique. By finely tuning the deposition sequence and other conditions, Ru and Co atoms are sequentially and accurately deposited onto pre-dispersed Pt single atoms, resulting in a well-defined triple-atom configuration. The incorporation of Co atoms plays a critical role in modulating the local coordination environment and electronic structure of both Pt and Ru atoms, thereby optimizing the overall catalytic landscape. This tailored atomic arrangement facilitates enhanced charge redistribution and synergistic metal–metal interactions, which are essential for boosting catalytic performance. Compared to their Pt SAs and Pt-Ru DAs counterparts, the Pt-Ru-Co TAs demonstrate significantly improved activity and stability, highlighting the advantages of multi-metal atom engineering at the atomic scale.

## Limitations

This protocol offers a reliable method for the precise construction of Pt-Ru-Co TAs catalyst. However, several limitations must be considered. ALD is highly sensitive to the substrate material, as the quality of NCNTs directly influences both the optimal deposition conditions and the catalytic performance of the final material. Even under carefully optimized conditions, the proportion of the desired Pt-Ru-Co triple-atom configuration remains limited, accounting for about 60% of the total metal species. The remainder consists of single atoms, dual atoms, and small clusters. Such structural heterogeneity is difficult to fully eliminate during multi-atom deposition and remains a common challenge in the development of atomically dispersed catalysts.

## Troubleshooting

### Problem 1

The prepared NCNTs substrate has some nanoparticles of Fe on the surface of nanotubes.

### Potential solution

Prolong the washing time of NCNTs. If necessary, use ultrasound or heating to aid in the removal of Fe nanoparticles formed during the synthesis process. To ensure consistent substrate quality, the same cleaning conditions should be applied in repeated experiments.

### Problem 2

The vacuum during ALD is low. A high vacuum is crucial during ALD to reduce the chance of cluster or nanoparticle formation. Low vacuum conditions can promote chemical vapor deposition (CVD)-like growth, leading to aggregation rather than isolated atom deposition.

### Potential solution

Regularly check and replace the vacuum oil of the mechanical pump. Inspect the airtightness of the ALD reaction chamber and replace the sealing O-rings as needed. Additionally, ensure the airtightness of the connection interfaces between the precursor cylinders and the ALD pipeline to prevent leakage and maintain stable deposition conditions.

### Problem 3

Precursor vapor pressure is too low when running the deposition recipe.

### Potential solution

Before deposition, heat the precursor and keep it for 30 min. If the precursor vapor pressure remains low during the pulse process, refill the precursor cylinder.

### Problem 4

The ALD chamber and pipelines are not clean and are contaminated by other precursors.

### Potential solution

Before starting the deposition and after completing it, heat the pipeline to the required temperature for the deposition reaction. Then, pulse N_2_ for 50 cycles to remove any residual precursor and clean the chamber and pipeline.

### Problem 5

In the HER reaction, bubbles appear on the catalyst surface, affecting mass transfer.

### Potential solution

Place a magnetic stir bar in the three-necked flask used for the electrocatalytic reaction and secure the flask on a magnetic stirrer. Stir the solution at an appropriate speed—sufficient to remove gas bubbles, but not so fast as to accelerate the detachment of the catalyst from the electrode surface. Ensure that the same stirring speed is maintained throughout the testing of all comparative catalysts to ensure consistency.

### Problem 6

The catalyst loaded on the glassy carbon electrode is uneven and easy to fall off in a short test time.

### Potential solution

When preparing the electrode solution, apply ultrasonic treatment for no less than 30 min. The ultrasonic treatment time can be extended depending on the dispersion of the solution to ensure uniformity. Avoid adding excessive electrode solution to the glassy carbon electrode to prevent it from spilling over the electrode surface. The drying temperature of the electrode should be between 50–60°C to avoid excessive heat, which can cause the catalyst layer to crack. Additionally, the catalyst loaded on the glassy carbon electrode should be kept moderate; excessive loading may cause the catalyst to harden and detach from the surface.

### Problem 7

The pH of the electrolyte solution is changed and improper.

### Potential solution

The actual pH value of the electrocatalytic solution directly affects the reaction rate of the HER and HOR. The appropriate pH of the solution should be maintained by using a freshly prepared electrolyte solution. The electrolyte cannot be stored for more than 30 days.

## Resource availability

### Lead contact

Further information and requests for resources should be directed to and will be fulfilled by the lead contact, Xueliang Sun (xsun9@uwo.ca).

### Technical contact

Technical questions on executing this protocol should be directed to and will be answered by the technical contact, Jingyan Zhang (jingyann.zhang@utoronto.ca).

### Materials availability

This study did not generate new unique reagents.

### Data and code availability

All experimental data included within the published article are available from the lead contact upon reasonable request.

## Acknowledgments

This work was financially supported by the 10.13039/100014717National Natural Science Foundation of China (nos. 22472101, 22075203, and 22279079), Guangdong Science and Technology Department Program (2021QN02L252 and 2023A1515010021), Research Team Cultivation Program of Shenzhen University (023QNT007), 10.13039/501100000038Natural Sciences and Engineering Research Council of Canada (NSERC), 10.13039/501100001804Canada Research Chair (CRC) Program, 10.13039/501100000196Canada Foundation for Innovation (CFI), and the 10.13039/501100004381University of Western Ontario. We also want to acknowledge Canadian Urban Transit Research and Innovation Consortium (CUTRIC) project and Ballard Power Systems, Inc.

## Author contributions

J.Z. designed and performed the experimental work, analyzed characterization results, and prepared the manuscript. Z.S. revised the manuscript carefully and provided suggestions. X.Y. carried out TEM test. Y.G. collected XAS data. Z.H. prepared the samples for TEM test and helped collect the results. N.C. analyzed the XANES and fitted Fourier-transformed EXAFS spectra. L.Z. guided the design of this work and revised the manuscript. X.S. supervised the overall project. All authors have approved the final version of the manuscript.

## Declaration of interests

The authors declare no competing interests.
